# Mutually exclusive antiproliferative effect of cell line‐specific *HOX* inhibition in epithelial ovarian cancer cell lines: SKOV‐3 vs RMUG‐S

**DOI:** 10.1111/jcmm.14993

**Published:** 2020-01-22

**Authors:** Miseon Kim, Dong Hoon Suh, Jin Young Choi, Seul Lee, Jae Ryul Bae, Kidong Kim, Jae Hong No, Yong Beom Kim

**Affiliations:** ^1^ Department of Obstetrics and Gynecology CHA Gangnam Medical Center CHA University School of Medicine Seoul Republic of Korea; ^2^ Department of Obstetrics and Gynecology Seoul National University Bundang Hospital Seongnam Republic of Korea; ^3^ Department of Obstetrics and Gynecology Chungbuk National University Hospital Cheongju Republic of Korea; ^4^ Department of Obstetrics and Gynecology College of Medicine Seoul National University Seoul Republic of Korea

**Keywords:** chemoresistance, HOX gene, ovarian cancer

## Abstract

We aimed to discover cell line‐specific overexpressed HOX genes responsible for chemoresistance and to identify the mechanisms behind HOX‐induced cell line‐specific chemoresistance in EOC. Ten HOX genes and eight EOC cell lines were tested for any cell line‐specific overexpression that presents a mutually exclusive pattern. Cell viability was evaluated after treatment with cisplatin and/or siRNA for cell line‐specific overexpressed HOX genes. Immunohistochemical (IHC) staining for HOXB9 was performed in 84 human EOC tissues. HOXA10 and HOXB9 were identified as cell line‐specific overexpressed HOX genes for SKOV‐3 and RMUG‐S, respectively. Inhibiting the expression of cell line‐specific HOX genes, but not of other HOX genes, significantly decreased cell viability. In SKOV‐3 cells, cell viability decreased to 46.5% after initial 10 µM cisplatin treatment; however, there was no further decrease upon additional treatment with HOXA10 siRNA. In contrast, cell viability did not significantly decrease upon cisplatin treatment in RMUG‐S cells, but decreased to 65.5% after additional treatment with HOXB9 siRNA. In both cell lines, inhibiting cell line‐specific HOX expression enhanced apoptosis but suppressed the expression of epithelial‐mesenchymal transition (EMT) markers such as vimentin, MMP9, and Oct4. IHC analysis showed that platinum‐resistant cancer tissues more frequently had high HOXB9 expression than platinum‐sensitive cancer tissues. HOXB9, which is overexpressed in RMUG‐S but not in SKOV‐3 cells, appeared to be associated with cell line‐specific platinum resistance in RMUG‐S. Inhibiting HOXB9 overexpression in RMUG‐S cells may effectively eliminate platinum‐resistant ovarian cancer cells by facilitating apoptosis and inhibiting EMT.

## 1 INTRODUCTION

An estimated 22 440 new cases of ovarian cancer were expected in the United States in 2017.^1^ There are a variety of histologic types of epithelial ovarian cancer (EOC), including serous, mucinous, endometrioid and clear cell carcinomas. Among these, mucinous tumours, which account for 10% of all primary EOCs, show poor prognosis compared with other subtypes in the advanced stage of disease^2^, ^3^; this has been mainly attributed to resistance to platinum‐based chemotherapy rather than tumour aggressiveness.^4^, ^5^ Nevertheless, all histologic subtypes of EOC have been treated with the same treatment strategy–maximal cytoreductive surgery followed by platinum‐based chemotherapy without consideration of the responsiveness to platinum.

HOX genes drive normal organogenesis through morphogenesis and terminal differentiation. Cheng et al showed that several HOX genes are differentially expressed in the fallopian tubes, uterus and vagina, but not in normal ovarian epithelium.^6^ They also suggested that the Müllerian‐like features of EOC are associated with the aberrant expression of HOX genes: *HOXA9* in serous carcinoma, and *HOXA10* and *HOXA11* in mucinous carcinoma. Different expression patterns and its prognostic value of HOX genes across distinct histologic subtypes of EOC have also been shown in several other studies.^6^, ^7^, ^8^, ^9^ However, the results of these studies were quite inconsistent and made it difficult to determine which HOX genes can be targeted to overcome chemoresistance in mucinous EOC.

This study aimed to discover any cell line‐specific overexpressed HOX genes that may be attributed to chemoresistance, as well as identify the mechanisms underlying HOX‐induced cell line‐specific chemoresistance in EOC cell lines.

## 2 METHODS

### 2.1 Cell culture and siRNA

SKOV‐3, a human ovarian cancer cell line of serous histology, was purchased from the American Type Culture Collection (ATCC). RMUG‐S, a human ovarian cancer cell line of mucinous histology, was obtained from the Japanese Collection of Research Bioresources Cell Bank (JCRB). siRNAs for human HOXA10 (5′‐ CCGGGAGCUCACAGCCAACUUUAAUUU −3′) and HOXB9 (5′‐ GGAAGCGAGGACAAAGAGAGGUU −3′) were synthesized by Genolution (Genolution Pharmaceutical Inc), and the control siRNA (sc‐37007) was purchased from Santa Cruz Biotechnology. The transient transfection experiment with the synthesized and control siRNAs was performed using Lipofectamine RNAi MAX™ according to the manufacturer's instruction (Invitrogen).

### 2.2 Western blot analysis and reverse transcription polymerase chain reaction (RT‐PCR)

Cells were lysed, and proteins were transferred to a polyvinylidene fluoride (PVDF) membrane. Membranes were incubated with anti‐HOXA10 (sc‐271954), anti‐HOXB9 (sc‐398500), anti‐E‐cadherin (#3195), anti‐Vimentin (#5741), anti‐MMP9 (#3852), anti‐SOX2 (#2748), anti‐Nanog (#3580), anti‐Oct4 (#2750) and anti‐alpha‐tubulin (sc‐5286) antibodies.

Cellular RNA was extracted from cells using the TRIzol reagent according to the manufacturer's instructions. Complementary DNA (cDNA) was synthesized from 2 µg of RNA using a reverse transcription kit (Promega) and each primer pair (Yingjun Biotechnology Corporation).

### 2.3 Annexin V‐fluorescein isothiocyanate (FITC) by flow cytometry

Annexin V‐FITC assay was carried out using the FITC Annexin V Apoptosis Detection Kit (BD Bioscience) according to the protocols provided. Flow cytometric analysis was performed using a FACS Calibur (BD Bioscience) flow cytometer, by analysing at least 10 000 cells per sample.

### 2.4 Terminal deoxynucleotidyl transferase‐mediated digoxigenin‐dUTP‐biotin nick‐end labelling (TUNEL) assay

The cells were transfected with either *HOXA10* or *HOXB9* siRNA for 72 hours. TUNEL was subsequently performed using the In Situ Cell Death Detection Kit (Roche) according to the manufacturer's instructions.

### 2.5 Wound healing migration assay and transwell invasion assay

The cells, which were transfected with *HOXA10* or *HOXB9* siRNA, were incubated overnight or until a monolayer formed. The monolayers were scratched with a 200‐µL sterile pipette tip and then washed with media to remove the detached cells and debris.

Cell invasion was measured in a transwell chamber. In brief, 2 × 10^5^ cells were added to each transwell invasion chamber coated with 1 mg/mL Matrigel (reconstituted basement membrane; BD Biosciences). After transfection with either *HOXA10* or *HOXB9* siRNA, the remaining cells on the membrane were fixed for 10 minutes in methanol, stained with 1% crystal violet solution, and then washed with PBS.

### 2.6 Immunohistochemistry

Immunohistochemical (IHC) staining of ovarian cancer tissues from an 84‐tissue microarray (TMA) was approved by the Institutional Review Board. The TMA was established using tissue from women who underwent surgery for the treatment of EOC between February 2000 and November 2009. Intensity of *HOXB9* nuclear staining was graded: negative, weak (1+), moderate (2+) or strong (3+). IHC scoring was performed according to the following criteria by MK and JYC (Figure 2A‐D): IHC score 0: samples with negative or equivocal staining, or <50% tumour cells with weak or combined moderate staining; IHC score 1:50% or more tumour cells with weak or combined weak and moderate staining, but <50% tumour cells with moderate or combined moderate and strong staining; IHC score 2:50% or more tumour cells with moderate or combined moderate and strong staining, but <50% tumour cells with strong staining; and IHC score 3:50% or more tumour cells with strong staining. IHC scores 2 and 3 were considered ‘high expression’.

### 2.7 Statistical analyses

Data are presented as means with standard deviations. When comparing between two groups, Student's *t* test was applied. Progression‐free survival (PFS) and overall survival (OS) were evaluated using the Kaplan‐Meier method. Statistical significance was taken as *P* < .05.

## 3 RESULTS

**Figure 1:**
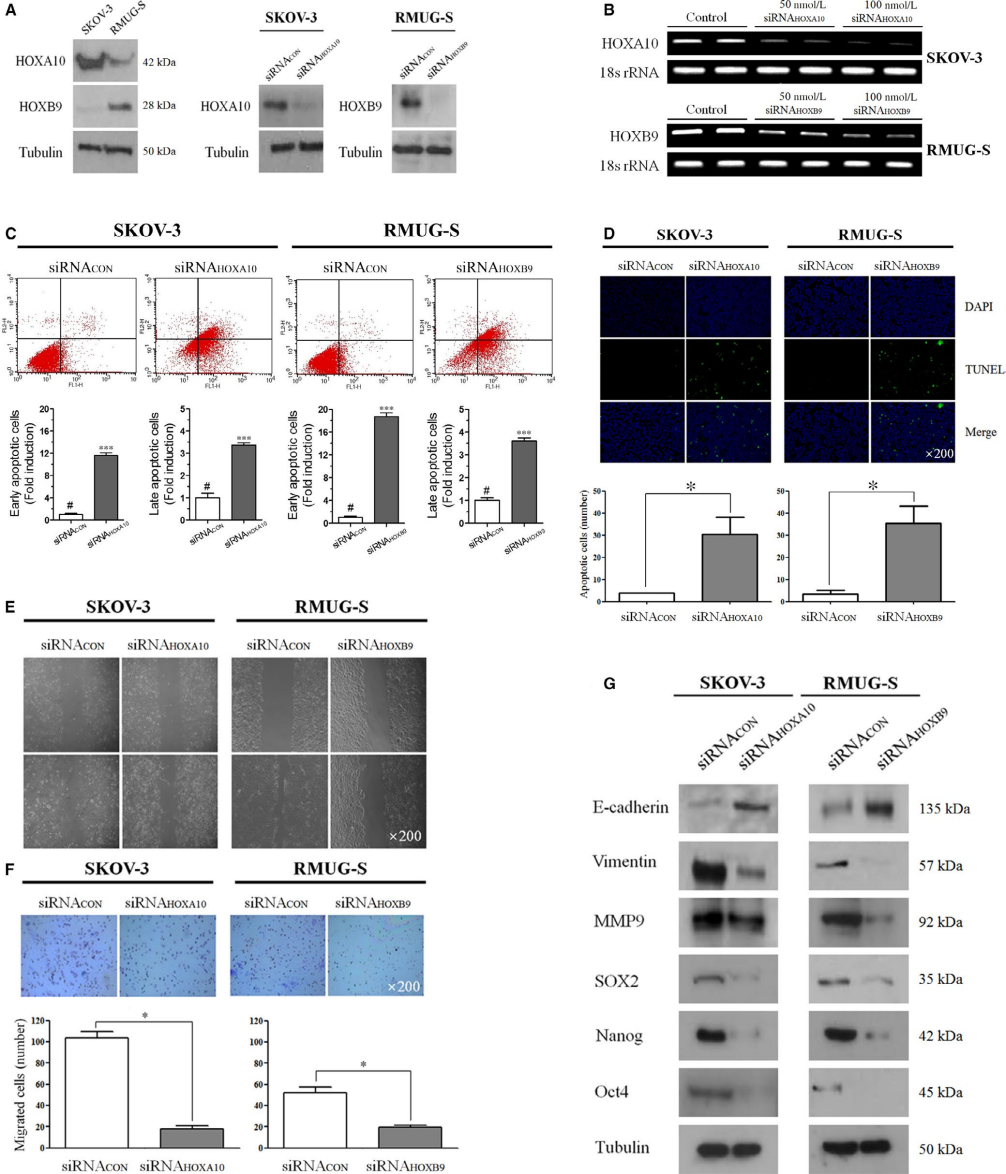
(A) Western blot analysis and (B) reverse transcription polymerase chain reaction (RT‐PCR) for *HOXA10* and *HOXB9* expression in SKOV‐3 and RMUG‐S cell lines with/without siRNA. (C) Early and late apoptotic cells after treatment with 50 nmol/L *HOXA10* and *HOXB9* siRNAs for 48 h in each cell line (D) images of TUNEL assay after treatment with corresponding siRNAs to each cell line (E) Wound healing migration assay, (F) transwell invasion assay and (G) Western blot analysis after treatment with corresponding HOX siRNAs in each cell line

Among the ten *HOX* genes whose expression levels were tested in eight EOC cell lines, *HOXA10* in SKOV‐3 and *HOXB9* in RMUG‐S were identified to have cell line‐specific overexpression with a mutually exclusive pattern between the two cell lines (Figure S1). *HOXA10* and *HOXB9* showed selectively high levels of expression in SKOV‐3 and RMUG‐S cell lines, respectively. *HOXA10* and *HOXB9* expression was inhibited by treatment with 50 nmol/L siRNA for the corresponding HOX genes, as observed through Western blotting. (Figure 1A) Upon RT‐PCR analysis, we also found that the mRNA expression levels of *HOXA10* and *HOXB9* were inhibited by treatment with siRNA in a dose‐dependent manner (Figure 1B). We performed Annexin V‐FITC (Fig. IC) and TUNEL (Figure 1D) assays to confirm the apoptotic effect of the siRNAs targeting each HOX gene. The number of apoptotic cells significantly increased after treatment with *HOXA10* siRNA for 72 hours in SKOV‐3, compared with those treated with a non‐targeting siRNA control. Similarly, *HOXB9* siRNA induced apoptosis in RMUG‐S.

The 24‐hour wound healing assay with *HOXA10* and *HOXB9* siRNA treatments showed decreased migration in SKOV‐3 and RMUG‐S cells, respectively (Figure 1E). In addition, the 72‐hour transwell invasion assay showed that the invading SKOV‐3 and RMUG‐S cells were significantly decreased when the cells were treated with *HOXA10* (17.5% vs 103.5%, *P* = .004) and *HOXB9* siRNAs (19.5% vs 52.0%, *P* = .017), respectively, compared with those treated with non‐targeting siRNA controls (Figure 1F). In both cell lines treated with their corresponding siRNAs, the expression levels of EMT‐related proteins including vimentin, MMP‐9, SOX2, NANOG and Oct4 decreased, while the expression level of E‐cadherin increased compared with those treated with their corresponding siRNA controls (Figure 1G).

**Figure 2:**
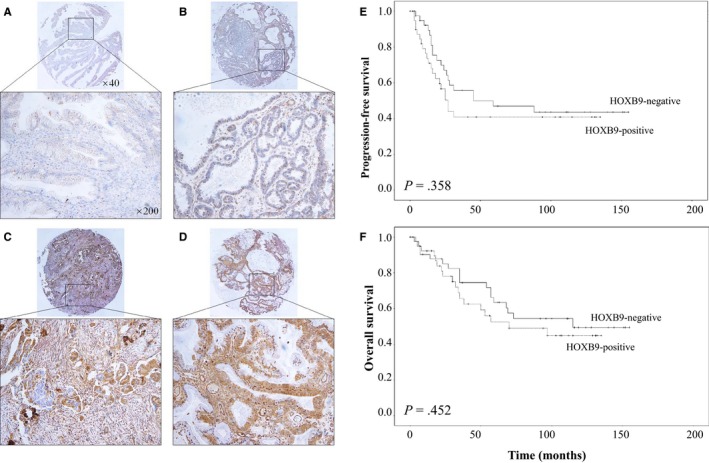
Image of immunohistochemical staining score (A) 0, (B) 1, (C) 2 and (D) 3 for *HOXB9* in ovarian cancer tissues in ×40 and ×200 magnifications. (E) Progression‐free survival and (F) overall survival graphs by Kaplan‐Meier methods according to the degree of *HOXB9* expression

The median follow‐up period of the 84 EOC patients was 55 months (1‐155 months). High expression of *HOXB9* was found in 40 (47.6%) female patients. Unlike the results observed in the cell lines, high *HOXB9* expression was not associated with mucinous histology in EOC patients (22.5% vs 18.2%, *P* = .623; Table S1). In the 70 patients who received platinum‐based chemotherapy after surgery, 34 (48.6%) EOC tissues highly expressed *HOXB9*. Resistance to platinum was more frequent in women with EOC tissues that exhibited high *HOXB9* expression (13/34 [38.2%] vs 5/36 [13.9%], *P* = .020). However, high HOXB9 expression was not associated with 5‐year PFS (47.0% vs 40.9%, *P* = .358) and OS (63.4% vs 52.5%, *P* = .452; Figure 2E, 2).

## 4 DISCUSSION

Apoptosis escape and EMT have been considered to be key processes in chemoresistance according to previous studies.^10^, ^11^ Chemotherapeutic agents generally induce tumour regression through apoptosis; however, the dysregulation of such apoptotic processes can lead to the increased expression of EMT‐inducing factors and result in the failure of chemotherapy. In this study, we evaluated whether the chemoresistance in an EOC cell line could be reversed by inhibiting a specific gene. This concept was derived from how the Müllerian‐like features in EOC were associated with the aberrant expression of HOX genes depending on the histologic type.^6^, ^7^, ^8^ Our findings suggest that *HOXB9* may contribute to platinum resistance in RMUG‐S by promoting apoptosis escape, as well as EMT.

There were only a few studies that reported on the impact of HOX expression on chemoresistance or prognosis in EOCs.^12^, ^13^, ^14^ The present study, to the best of our knowledge, is the first study that identified *HOXB9* to possibly be responsible for chemoresistance in a mucinous EOC cell line, RMUG‐S. Demonstration of the association of *HOXB9* high expression and platinum resistance using clinical data and human tissue was also a strength of our study; although, we were unable to show any independent survival impact due to *HOXB9* high expression. This may be attributed to the small sample number of our study, as well as to the use of a small fractionated TMA block instead of whole ovarian cancer tissues.

*HOXB9*, which was found to be overexpressed in RMUG‐S but not in SKOV‐3 cells, appeared to be associated with cell line‐specific platinum resistance. Inhibiting *HOXB9* overexpression in RMUG‐S cells can effectively kill platinum‐resistant ovarian cancer cells by facilitating apoptosis and inhibiting EMT. Further in vivo studies and clinical trials are necessary to develop an individualized strategy for effectively controlling chemoresistance in mucinous type EOCs.

## CONFLICTS OF INTEREST

The authors confirm that there are no conflicts of interest.

## AUTHOR'S CONTRIBUTION

M. Kim, D. H. Suh and Y. B. Kim conceived and designed the study; M. Kim, D. H. Suh, J. Y. Choi, S. Lee and J. R. Bae performed the data analysis and interpreted the results; M. Kim wrote the manuscript and K. Kim, J. H. No and Y. B. Kim reviewed and revised the manuscript. All authors reviewed the final manuscript.

## Data Availability

The data that support the findings of this study are available from the corresponding author upon reasonable request.
