# Supplementary figures and images for: Mutually exclusive antiproliferative effect of cell line‐specific HOX inhibition in epithelial ovarian cancer cell lines: SKOV‐3 vs RMUG‐S
Source: J Cell Mol Med. 2020 Jan 22;24(5):3246–51. doi: 10.1111/jcmm.14993 (PMC7077590; doi:10.1111/jcmm.14993)

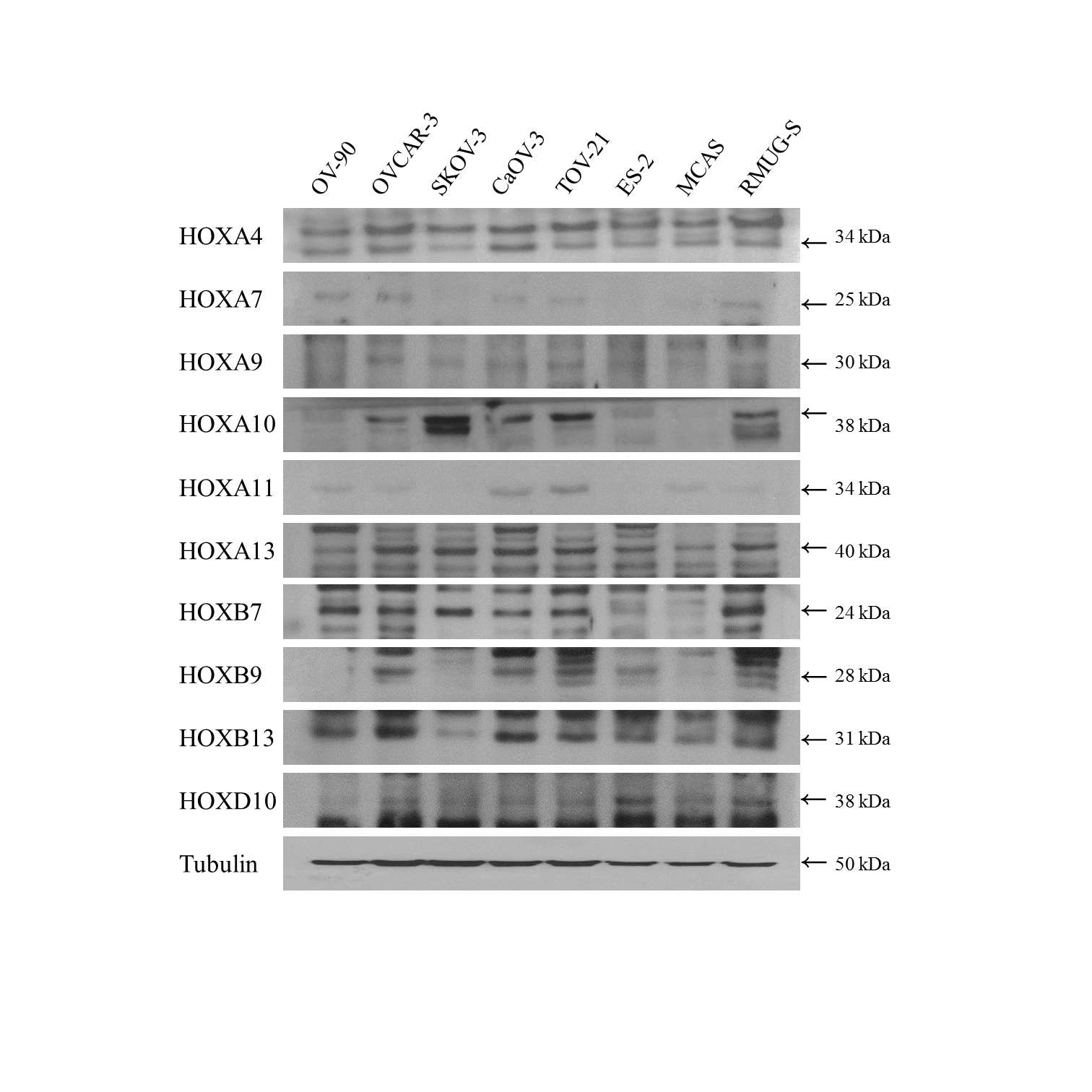

Supplement: Supplementary file 1 [file JCMM-24-3246-s001.tif]
