# Supplementary material for: Mutually exclusive antiproliferative effect of cell line‐specific HOX inhibition in epithelial ovarian cancer cell lines: SKOV‐3 vs RMUG‐S
Source: J Cell Mol Med. 2020 Jan 22;24(5):3246–51. doi: 10.1111/jcmm.14993 (PMC7077590; doi:10.1111/jcmm.14993)
Supplement: Supplementary file 2 [file JCMM-24-3246-s002.docx]

| **Supplementary Table 1.** Comparisons of histologic types and the response to platinum according to the degree of HOXB9 expression by immunohistochemical staining analysis in 84 EOC patients | | | | |
| --- | --- | --- | --- | --- |
|  | Total *n* | Low expression of HOXB9  (*n* = 44) | High expression of HOXB9  (*n* = 40) | *P* value |
| **Histology** | |  |  | 0.623 |
| Serous type | 67 | 36 (53.7) | 31 (46.3) |  |
| Mucinous type | 17 | 8 (47.1) | 9 (52.9) |  |
| **Response to platinum**† | |  |  | 0.020 |
| Sensitive | 52 | 31 (59.6) | 21 (40.4) |  |
| Resistant | 18 | 5 (27.8) | 13 (72.2) |  |
| IHC score 0, samples with negative or equivocal staining, or <50% tumor cells with weak (1+) or combined moderate (2+) staining; IHC score 1, 50% or more tumor cells with weak (1+) or combined weak (1+) and moderate (2+) staining, but less 50% tumor cells with moderate (2+) or combined moderate (2+) and strong (3+) staining; IHC score 2, 50% or more tumor cells with moderate (2+) or combined moderate (2+) and strong (3+) staining, but less than 50% tumor cells with strong (3+) staining; IHC score 3, 50% or more tumor cells with strong (3+) staining. IHC score 2 and 3 were considered as ‘high expression’.  †in 70 patients received platinum-based chemotherapy after surgery  EOC, Epithelial ovarian cancer | | | | |
